# 2644. Presence of Respiratory Viruses in the Upper Respiratory Tract of Febrile Infants ≤90 Days Old is Associated with Lower Prevalence of Invasive Bacterial Infection

**DOI:** 10.1093/ofid/ofad500.2256

**Published:** 2023-11-27

**Authors:** Erin G Nicholson, Tess Stopczynski, Leila C Sahni, Vasanthi Avadhanula, Pedro A Piedra, Justin Z Amarin, James Chappell, Eileen J Klein, Janet A Englund, John V Williams, Marian G Michaels, Geoffrey A Weinberg, Peter G Szilagyi, Jennifer E Schuster, Rangaraj Selvarangan, Mary A Staat, Elizabeth P Schlaudecker, Veronica Burkel, Heidi L Moline, Natasha B Halasa, Julie A Boom

**Affiliations:** Baylor College of Medicine, Houston, Texas; Vanderbilt University Medical Center, Nashville, TN; Baylor College of Medicine and Texas Children’s Hospital, Houston, Texas; Baylor College of Medicine, Houston, Texas; Baylor College of Medicine, Houston, Texas; Vanderbilt University Medical Center, Nashville, TN; Vanderbilt University Medical Center, Nashville, TN; University of Washington School of Medicine, Seattle, Washington; Seattle Children’s Hospital, Seattle, Washington; University of Pittsburgh, Pittsburgh, Pennsylvania; UPMC Children's Hospital of Pittsburgh, Pittsburgh, Pennsylvania; University of Rochester School of Medicine & Dentistry, Rochester, NY; UCLA School of Medicine, Agoura Hills, California; Children’s Mercy Kansas City, Kansas City, Missouri; Children’s Mercy Kansas City, Kansas City, Missouri; Cincinnati Children’s Hospital Medical Center, Cincinnati, Ohio; Cincinnati Children's Hospital Medical Center, Cincinnati, Ohio; Eagle Health Analytics / CDC, Atlanta, Georgia; Centers for Disease Control and Prevention, Atlanta, Georgia; Vanderbilt University Medical Center, Nashville, TN; Texas Children’s Hospital, Houston, Texas

## Abstract

**Background:**

Infants ≤ 90 days old are at increased risk of severe disease from both viral and bacterial pathogens. Differentiating between these two etiologies has long presented a diagnostic challenge and has resulted in many risk algorithms to determine which infants are infected by bacteria and require antibiotics. The increased availability of rapid molecular diagnostics presents an opportunity to refine these algorithms. While the prevalence of urinary tract infection in the presence of a respiratory virus has been described, large-scale studies with comprehensive viral testing describing the prevalence of invasive bacterial infection (IBI), defined as bacteremia or bacterial meningitis, are lacking.

**Methods:**

The CDC’s New Vaccine Surveillance Network (NVSN) enrolls children < 18 years old with acute respiratory illness in the emergency department or inpatient setting of 7 hospitals. Upper respiratory specimens are collected and tested for respiratory viruses by real-time PCR. In this analysis respiratory viral positivity is described in febrile and non-febrile infants ≤ 90 days old. We then compared the prevalence of IBI (based on blood and CSF cultures obtained for clinical testing) among febrile infants with and without a respiratory virus detected. IBIs were defined as a positive blood or CSF culture with bacteria unlikely to be a contaminant.

**Results:**

From December 2016 to March 2020, 3,731 infants (*n*=2,138 febrile, *n*=1,593 non-febrile) were enrolled. Respiratory viral positivity was 68% for febrile infants and 78% for non-febrile infants. Among febrile infants, 68% (*n*=1,461) had either a blood or CSF culture obtained; 3% (*n*=41) had an IBI. Febrile infants in whom a respiratory virus was detected, were significantly less likely to have an IBI (OR: 0.26 [95% CI: 0.13–0.52]) than febrile infants with negative viral testing (Table).

**Figure:**
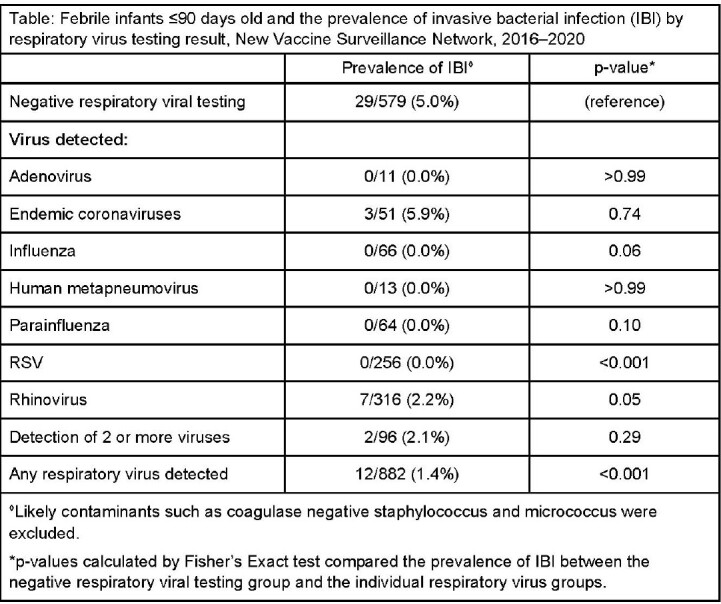

**Conclusion:**

Detection of a respiratory virus in febrile infants ≤90 days is associated with significantly lower odds of an IBI (bacteremia or bacterial meningitis). These findings may help develop future risk algorithms for bacterial infection in febrile infants aged ≤90 days aiming to decrease unnecessary antibiotic use in this population.

**Disclosures:**

**Erin G. Nicholson, MD, MS**, Blue Lake Biotechnology Inc.: Grant/Research Support|Novavax: Advisor/Consultant **Pedro A. Piedra, MD**, Ark Bioscience: Advisor/Consultant|Ark Bioscience: Grant/Research Support|GSK: Grant/Research Support|Icosavax: Advisor/Consultant|Icosavax: Grant/Research Support|Mapp Biologics: Grant/Research Support|Meissa Vaccines: Grant/Research Support|Moderna: Advisor/Consultant|Novavax: Advisor/Consultant|Novavax: Grant/Research Support|Sanofi-Pasteur: Grant/Research Support|Shionogi: Advisor/Consultant|Shionogi: Grant/Research Support|Takeda: Advisor/Consultant **Janet A. Englund, MD**, Ark Biopharma: Advisor/Consultant|AstraZeneca: Advisor/Consultant|AstraZeneca: Grant/Research Support|GlaxoSmithKline: Grant/Research Support|Meissa Vaccines: Advisor/Consultant|Merck: Grant/Research Support|Moderna: Advisor/Consultant|Moderna: Grant/Research Support|Pfizer: Advisor/Consultant|Pfizer: Grant/Research Support|Sanofi Pasteur: Advisor/Consultant **John V. Williams, MD**, Merck: Grant/Research Support|Quidel: Board Member **Marian G. Michaels, MD, MPH**, Merck: Grant/Research Support|Viracor: Grant/Research Support **Geoffrey A. Weinberg, MD**, Merck & Co: Honoraria **Rangaraj Selvarangan, BVSc, PhD, D(ABMM), FIDSA, FAAM**, Abbott: Honoraria|Altona Diagnostics: Grant/Research Support|Baebies Inc: Advisor/Consultant|BioMerieux: Advisor/Consultant|BioMerieux: Grant/Research Support|Bio-Rad: Grant/Research Support|Cepheid: Grant/Research Support|GSK: Advisor/Consultant|Hologic: Grant/Research Support|Lab Simply: Advisor/Consultant|Luminex: Grant/Research Support **Mary A. Staat, MD, MPH**, CDC: Grant/Research Support|Cepheid: Grant/Research Support|Merck: Grant/Research Support|NIH: Grant/Research Support|Pfizer: Grant/Research Support|Up-To-Date: Honoraria **Elizabeth P. Schlaudecker, MD, MPH**, Pfizer: Grant/Research Support|Sanofi Pasteur: Advisor/Consultant **Natasha B. Halasa, MD, MPH**, Merck: Grant/Research Support|Quidell: Grant/Research Support|Quidell: donation of kits|Sanofi: Grant/Research Support|Sanofi: vaccine support

